# Dual Amplified Spontaneous Emission and Lasing from Nanographene Films

**DOI:** 10.3390/nano10081525

**Published:** 2020-08-04

**Authors:** Rafael Muñoz-Mármol, Víctor Bonal, Giuseppe M. Paternò, Aaron M. Ross, Pedro G. Boj, José M. Villalvilla, José A. Quintana, Francesco Scotognella, Cosimo D’Andrea, Samim Sardar, Guglielmo Lanzani, Yanwei Gu, Jishan Wu, María A. Díaz-García

**Affiliations:** 1 Departamento de Física Aplicada and Instituto Universitario de Materiales de Alicante, Universidad de Alicante, 03080 Alicante, Spain; rafa.marmol@ua.es (R.M.-M.); victor.bonal@ua.es (V.B.); jmvs@ua.es (J.M.V.); 2 Center for Nano Science and Technology, Istituto Italiano di Tecnologia, Via G. Pascoli 70/3, 20133 Milano, Italy; giuseppe.paterno@iit.it (G.M.P.); francesco.scotognella@polimi.it (F.S.); cosimo.dandrea@polimi.it (C.D.); samim.sardar@iit.it (S.S.); 3 Physics Department, Politecnico di Milano, Piazza L. da Vinci 32, 20133 Milano, Italy; aaronmichael.ross@polimi.it; 4 Departamento Óptica, Farmacología y Anatomía and Instituto Universitario de Materiales de Alicante, Universidad de Alicante, 03080 Alicante, Spain; p.boj@ua.es (P.G.B.); ja.quintana@ua.es (J.A.Q.); 5 Department of Chemistry, National University of Singapore, 3 Science Drive 3, Singapore 117543, Singapore; a0134755@u.nus.edu

**Keywords:** organic lasers, amplified spontaneous emission, nanographenes

## Abstract

Chemically synthesized zigzag-edged nanographenes (NG) have recently demonstrated great success as the active laser units in solution-processed organic distributed feedback (DFB) lasers. Here, we report the first observation of dual amplified spontaneous emission (ASE) from a large-size NG derivative (with 12 benzenoid rings) dispersed in a polystyrene film. ASE is observed simultaneously at the 685 and 739 nm wavelengths, which correspond to different transitions of the photoluminescence spectrum. Ultrafast pump-probe spectroscopy has been used to ascertain the underlying photophysical processes taking place in the films. DFB lasers, based on these materials and top-layer nanostructured polymeric resonators (i.e., one or two-dimensional surface relief gratings), have been fabricated and characterized. Lasers emitting close to either one of the two possible ASE wavelengths, or simultaneously at both of them, have been prepared by proper selection of the resonator parameters.

## 1. Introduction

Conjugated organic materials have been demonstrated to be useful for a variety of optoelectronics applications, e.g., organic light-emitting diodes, organic solar cells, or organic field-effect transistors. Particularly, great effort has been devoted to organic active materials for laser applications [1,2]. The excellent light–matter interaction taking place in semiconducting and/or photoluminescent materials, along with their flexibility for integration with other technologies through wet-fabrication techniques [3], place them as a leading technology for the next generation of laser devices. Part of the interest of these materials corresponds to the development of bottom–up fabrication techniques in organic chemistry [4]. Such techniques allow controlling their optoelectronic properties through molecular design with outstanding precision and reproducibility. However, obtaining strong performance from all organic solution-processed devices (i.e., both resonator and active material) simultaneously for the various aspects that are important for applications, such as low pump power requirements, large operational stability, or systematic color tuning, remains a challenge.

Graphene is a promising organic material for the fabrication of integrated devices that have possibilities in optoelectronics [5] and spintronics [6]. Although initially, its vanishing electronic gap prevented its use on semiconducting applications, nowadays, a gap can be opened using different techniques. Initial attempts by oxidation (in graphene oxides) [7], by π-stacking of graphene layers (in few-layer graphenes) [8], or by curvature (in curved graphenes) [9] succeeded, but they were demonstrated to be insufficient for gain amplification [10,11]. Conversely, attempts to structural patterning graphene into nanostructures (nanographenes, NGs) [12] have been demonstrated as fit for such applications. This approach relies on the quantum confinement of the electronic wave function, which decidedly depends on the size and edge structure of the NG compound [13]. To this purpose, the organic chemistry bottom–up approach has been applied to NG production with greater success than previously used top–down techniques, which lack control on the final product. With this step forward, amplified spontaneous emission (ASE) was reported for the first time in polystyrene (PS) films doped with an NG derivative [14,15], together with its all-optical switching demonstration [16]. Subsequently, ASE was reported in a series of three NGs of increasing size (FZ*n*, with *n* = 1, 2, 3), which were profited to implement, for the first time, NG-based distributed feedback (DFB) lasers [12].

Over time, ingenious solutions for multicolor emission have been put forward, e.g., active compounds mixtures [17,18,19,20,21,22] or tricky device architectures [23], all consisting of intricate solutions leading to complex systems. Conversely, compounds with dual ASE represent a possible approach toward this goal. To date, just some materials with dual ASE have been reported in the literature. Among these, most correspond to materials in liquid solution, as is the case of coumarin derivatives [24,25]. These molecules suffer twisted intramolecular charge transfer, which consists of a twisted version of the molecules with different energy states, so their dual ASE emission corresponds to the superposition of the ASE emission of each molecular species. Similarly, dual ASE in liquid solutions of polyfluorene (PFO), conjugated polymers (MEH-PPV), and copolymers (PFO-*co*-MEH-PPV) has been ascribed to the coexistence of monomer, dimer, and excimer states [26,27,28,29,30,31]. Apart, dual ASE in the liquid solution of some oligo (phenylene vinylene) derivatives has been attributed to emission from the main transition and its vibronic replica [32]. Further, fewer cases of dual ASE have been reported to occur in solid films, and most of them correspond to PFO derivatives. In them, the dual ASE is attributed to the coexistence of two phases of the polymer, which can emit simultaneously [33,34], and/or to the coexistence of a monomer and excimer [35]. Aside from these, dual ASE was found in a bifluorene derivative single crystal, which was related to the emission from two different vibronic replicas [36].

Here, we report the first evidence of dual ASE emission from an NG compound (i.e., FZ3) dispersed in a PS film. The two ASE peaks appear at 685 and 739 nm, which places FZ3 as the NG emitting farther in the near-infrared region up to date. The underlying photophysical processes are analyzed by ultrafast pump-probe spectroscopy. It is also demonstrated that both gain bands are operative for lasing applications by the preparation of DFB devices with different periods. Finally, a 2D DFB laser emitting simultaneously from both gain bands is reported as a proof of concept.

## 2. Materials and Methods

### 2.1. Synthesis

FZ3 was synthesized following a multi-step process involving Pd-catalyzed C–C coupling, Friedel–Crafts-type alkylation and oxidative dehydrogenation. This synthesis has been previously described in detail in our previous work [37].

### 2.2. Sample Preparation

PS films doped with FZ3 (1 wt %) were spin-coated on quartz substrates using toluene (Merck KGaA, Darmstadt, Germany) as solvent. A film thickness (*h_f_*) of around 600 nm was determined through the interference pattern observed in the non-absorbing region of the absorption spectrum [38]. Such films constitute planar waveguides that support just one transversal electric mode (TE_0_) and one transversal magnetic mode (TM_0_), both propagating with a high confinement factor (Γ approximately 90%) [39]. This minimizes waveguide losses and therefore the ASE threshold [40].

DFB lasers based in these films were fabricated following three steps: First, the negative photoresist dichromated gelatin (DCG), incorporating 35 wt % of ammonium dichromate as a sensitizer, was spin-coated over the FZ3-doped film from a hot water solution (2.2 wt %, 40 °C), resulting in a 100 nm film. Then, one- or two-dimensional gratings were recorded using a holographic lithography (HL) technique. For it, interference patterns were generated with an Argon laser (*λ* = 364 nm) using a Lloyd’s setup where a mirror is attached with a 90° angle to the sample holder [41]. The grating period was adjusted by changing the incident angle. The intensities of direct and reflected beams were approximately equal, with an average exposure of 45 mJ/cm^2^, which guarantee the maximum contrast [42]. Backward reflections were prevented by attaching an absorbing plate with an index matching liquid to the back of the sample. Single and double exposition was used for the one and two-dimensional gratings, respectively. Finally, the DCG layer was desensitized in a cool water bath (10 °C), and the relief gratings were obtained through a dry development with an exposition to oxygen plasma (Diener Zepto).

### 2.3. Optical Characterization

The absorption (ABS) and photoluminescence (PL) spectra of the doped films were determined using a double-beam V-650 spectrometer (Jasco-Spain, Madrid, Spain) and a Horiba Nanolog fluorimeter (Horiba Italia SRL, Torino, Italy), respectively.

ASE measurements of the waveguide samples were performed using, as the excitation source, an Nd:YAG laser (LOTIS TII, Minsk, Belarus) with built-in optical parameter oscillator (OPO) providing conversion of the Nd:YAG third harmonic (355 nm, 10 Hz) to the pumping radiation beam (613 nm, 10 Hz). The pump wavelength (*λ_pump_*) was selected to match the first vibronic of the FZ3 steady-state absorption spectrum (at *λ* = 613 nm), just to avoid the superposition of the pump and the emitted light. For such a wavelength, the time duration of the pump pulses was 4.5 ± 0.1 ns. The energy of the pump beam was controlled with neutral density filters. This beam was collimated with an inverted telescope. Then, it was shaped over the sample surface as a narrow stripe (0.5 × 3.5 mm^2^) with a cylindrical lens and an adjustable slit. The guided PL light was collected from the sample’s edge with an optical fiber and analyzed with an USB2000+ UV-VIS fiber spectrometer (Ocean Optics, Ostfildern, Germany) with 1.3 nm spectral resolution. Plots of the linewidth (defined as the full width at half maximum, *FWHM*) and the output intensity (*I_out_*) versus the pump energy density, *E_pump_* were used to determine the ASE threshold (*E_th-ASE_*). This corresponds to the value at which the *FWHM* drastically decreases (and the *I_out_* increases). The DFB lasers were characterized using the same excitation source used for the ASE measurements and the same optical setup, but replacing the cylindrical lens and the slit by a spherical lens. Here, the beam impinged the sample surface at a 30° angle with respect to its normal direction, thus forming an elliptical spot over it (shorter axis radius of 0.5 mm). This configuration is used to spatially filter the pump beam from the emission. The emitted light is collected in a direction perpendicular to the device surface with an optical fiber and analyzed with the same spectrometer used for ASE characterization.

Ultrafast non-degenerate pump-probe measurements were performed with an amplified Ti:Sapphire laser (800 nm, 1 kHz and 2 mJ output). The pump wavelength (*λ_pump_*) was tuned to 618 nm with an optical parametric amplifier (OPA). The broadband probe (from 500 to 700 nm) was generated following two steps: first, the 800 nm was tuned to 1130 nm with an OPA; and second, it was focused into a YAG plate. The pump and probe beams were overlapped on the sample by focusing them within a 440 μm diameter spot, while the pump fluence was kept low (approximately 30 mJ/cm^2^) to avoid damaging the sample. The stimulated emission cross-section (*σ_SE_*) has been determined through Equation (1) [43]:

(1) $$\sigma_{SE}=\frac{\Delta T}{T}\frac{hc}{E_{pump}\lambda_{pump}}\frac{1}{\left(1-\exp\left(-\alpha h_{f}\right)\right)}\frac{1}{\left(1-R\right)}\;,$$

where ∆*T*/*T* is the transient absorption (TA) peak value, *α* is the absorption coefficient at *λ_pump_*, *R* is reflectivity of PS, *c* is the speed of light, and *h* is the Planck constant.

The fluorescence lifetime has been evaluated by using two time-resolved photoluminescence systems based on Time-Correlated Single Photon Counting (TCSPC) technique [44] and a streak camera [45], respectively. For TCSPC experiments, a supercontinuum laser (Fianium, Southampton, UK), emitting picosecond pulses at 40 MHz over the spectral range 480–1700 nm was used as the excitation source, and proper band-pass filters (500 nm and 620 nm) were used to select the excitation wavelengths. The emitted light was collected orthogonally to the excitation with a lens, and the excitation was filtered out with a long-pass filter. The filtered light was spatially dispersed by an imaging spectrometer (SP-2150i Princeton Instruments, Trenton, NJ, USA) and finally imaged onto a 16-channel photomultiplier (about 9 nm/channel) coupled to a TCSPC data acquisition board (Becker and Hickl, Berlin, Germany). The Instrumental Response Function (IRF) was about 200 ps wide (FWHM). For streak camera measurements, the excitation wavelength was 420 nm, and it was generated by the combination of a femtosecond Ti:Sapphire laser (Coherent, Santa Clara, CA, USA) at 80 MHz rep. rate, a home-made OPO, and a Second Harmonic Generation module, while the emitted light was collected by a streak camera coupled to a spectrometer. The Instrumental Response Function (IRF) was about 20 ps wide (FWHM). In both cases, the obtained time-resolved PL was fitted to a single exponential decay to determine the fluorescence lifetime (*τ_fl_*).

## 3. Results and Discussion

### 3.1. Optical and ASE Properties of FZ3 Films

**Figure 1:**
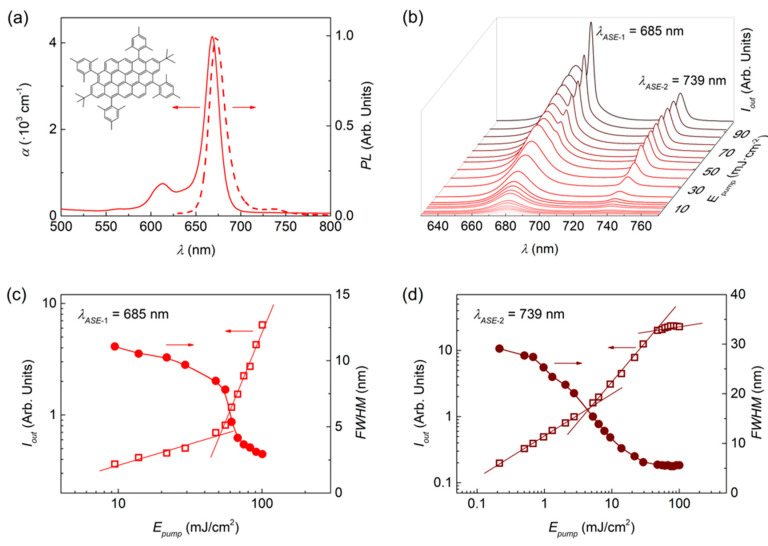
Optical and amplified spontaneous emission (ASE) properties of FZ3-doped polystyrene (PS) films. (**a**) Absorption (solid line, left axis) and photoluminescence (dashed line, right axis) spectrum of a FZ3 doped film. Chemical structure shown as inset; (**b**) Emission spectra at different pump energy densities (see legend), with ASE peaks appearing at *λ_ASE_*_-1_ = 685 nm and *λ_ASE_*_-2_ = 739 nm; (**c**,**d**) Output intensity (open squares, left axis) and emission linewidth, defined as the full width at half maximum, *FWHM* (solid circles, right axis) of these two narrowing peaks, respectively, as a function of the pump energy density, for ASE threshold determination. Lines are guides to the eye.

The optical and ASE properties of films containing FZ3 are described through results shown in Figure 1 and Table 1. The ABS and PL spectra (see Figure 1a), with main peaks at 668 and 676 nm, respectively, are mirror-like and display a small Stokes shift, which is typical of planar compounds. The PL spectrum shows a vibrational transition at around 740 nm, related to CC in-plane vibrational stretching modes [12]. Under a pulsed optical pump, ASE has been observed at two different wavelengths, 685 nm and 739 nm (see Figure 1b), corresponding to the main PL band (0′→0) and the first vibronic transition (0′→1), respectively. In a previous study of FZ3, only the ASE emission at the lowest wavelength was reported [12]. Figure 1c,d show the output intensity and linewidth, as a function of the pump energy density, for the ASE peaks at 685 and 739 nm, respectively. In both, a clear spectral narrowing at a given pump energy density (the ASE threshold, *E_th-ASE_*), accompanied by a change in the output intensity slope, can be seen. Noticeably, the threshold for the ASE peak at 739 nm (*E_th-ASE_* = 4.4 mJ/cm^2^) is significantly lower than that of the ASE peak at 685 nm (*E_th-ASE_* = 60 mJ/cm^2^). This is attributed to the larger reabsorption losses of the latter, which is due to the proximity of the absorption band. It should also be noted that the intensity of the 739 nm ASE peak saturates precisely at the ASE threshold of the 685 nm ASE peak (see Figure 1c). Thus, for pump intensities above this value, both ASE peaks can be seen simultaneously.

### 3.2. Transient Absorption of FZ3 Films

**Figure 2:**
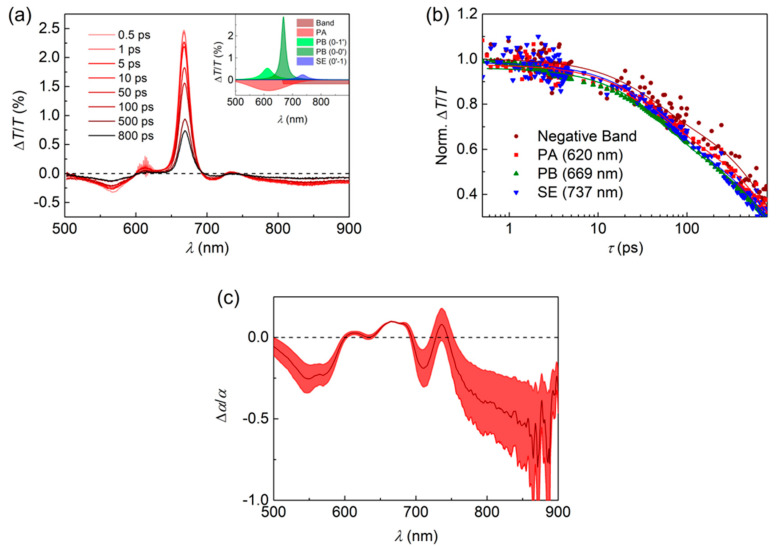
Transient absorption spectra for FZ3-doped PS films. (**a**) Transient absorption (TA) spectra obtained at a pump wavelength of 618 nm and energy density of 30 μJ/cm^2^ for a series of pump-probe delays (see legend). The inset represents the deconvolution of the 10 ps pump-probe delay curve into five different contributions, attributed to photobleaching (PB), photoinduced absorption (PA) or stimulated emission (SE); (**b**) Transient dynamics for the peaks at 620 nm, 669 nm, and 737 nm, attributed to PA, PB, and SE, respectively and for the negative band. Solid lines correspond to double exponential fits to the data; (**c**) Pump-induced absorption coefficient normalized to steady-state absorption coefficient (dark red line) and its error estimation (red area).

Figure 2 shows results obtained from TA spectroscopy studies, under a 618 nm pump wavelength, on FZ3-doped PS films. TA spectra for a series of pump-probe delays between 0.5 and 800 ps are shown in Figure 2a. The spectra present five main features, which can been deconvoluted, as seen in the inset for the 10 ps pump-probe delay spectrum. The main central peak at 669 nm and its side peak at 613 nm seem to correspond to photobleaching (PB), as they spectrally coincide with the main steady-state absorption peak (0→0′) and its vibronic replica (0→1′), respectively. The peak at 737 nm can be attributed to stimulated emission (SE), which occurs from the first vibronic transition (0′→1) of the emission spectrum. Finally, a global negative background extending across a wide range of wavelengths can be observed. This seems to have two contributions: a negative peak centered at 620 nm, which can be attributed to photoinduced absorption (PA); and a negative constant band, which starts at 666 nm and extends toward larger wavelengths, whose origin is not clear at the moment. As shown in Figure 2b, the transient dynamics of all these features are similar, as they correspond to processes that take place from the same energetic level. These dynamics show a double decay exponential with a dominant component of approximately 1.0 ns—three times shorter than its fluorescence lifetime (*τ_fl_* = 3.1 ± 0.3 ns). It is also interesting to analyze the pump-induced change in the absorption coefficient relative to the steady-state absorption coefficient (∆*α*/*α*, see Figure 2c). Here, the positive features correspond to gain bands. Particularly, two positive features of similar intensity appear, at approximately 680 nm and 737 nm, which correspond to the main transition (0′→0) and its first vibronic replica (0′→1), respectively. The presence of these two gain bands of similar intensity accounts for the dual ASE phenomenon observed in FZ3.

The origin of the double-gain band in FZ3-doped films can be identified from the confrontation of its TA with that of FZ1 and FZ2. In the supporting information, Figures S1a and S2a show respectively the TA spectra of FZ1 and FZ2-doped films for several pump-pulse delays between 0.5 and 800 ps. For these NGs, the TA spectrum corresponds with the convolution of various features as represented in the insets. Similarly to FZ3, the positive main peaks (at 447 and 547 nm, respectively for FZ1 and FZ2) and their left-side positive peaks are ascribed to PB from the ground state, and the positive right-side peaks correspond to SE from the first vibronic replica of the emission spectrum. For each NG, the deconvolution indicates just one negative peak (at 620 and 520 nm, respectively for FZ1 and FZ2) that is attributed to PA. Notice the missing negative band that was present in the TA spectrum of FZ3. For each NG, the transient features show similar dynamics (see Figures S1b and S2b). Again, as in the case of FZ3, these transients follow a double-decay exponential, which presents a dominant component of approximately 1.0 ns and 1.5 ns for FZ1 and FZ2, respectively. Intriguingly, all three NGs present similar transient absorption lifetimes, even though their fluorescence lifetimes differ from one another, see Figure S3 and Table S1. Their fluorescence lifetime values increase with the size of the NG, certainly, as a consequence of the electronic wave function delocalization. A larger difference between NGs appears among their ∆*α*/*α* spectra, as shown in Figures S1c and S2c. Here, the FZ1 spectrum presents a dominant gain peak at 482 nm, which coincides spectrally with its (0′→1) transition. In the case of FZ2, a considerable gain peak appears at 554 nm, coinciding with its (0′→0) transition, but again, the dominant gain peak at 594 nm coincides with its (0′→1) transition. For both NGs, ASE was reported at approximately 486 and 591 nm, coinciding respectively with the dominant gain peak of each NG [12]. Indeed, FZ3 shows two gain peaks of similar value; then, ASE emission can be observed from each of them. This change in the gain pattern is attributed to the negative band that appears in the TA spectrum of FZ3; it balances both gain bands by partially countering the one at 737 nm. Moreover, the effect of the negative band is recognized in the ASE threshold of the compound. The SE cross-section estimated for each NG shows similar values for the three within the error (approximately 10^−15^ cm^2^). However, FZ3 presents an ASE threshold that is at least 10 times larger, which is certainly affected by the superposition with the negative band.

Some hypotheses can be proposed regarding the origin of the negative band in the TA spectrum of FZ3. The full electronic structure of these NGs remains unknown beyond some time-dependent density functional theory calculations of their singlet states [37], which precludes a rigorous interpretation of the negative band. Fortunately, these NGs resemble closely the acenes, which have been thoroughly studied in the literature. It has been reported that large acenes show a sort of charge-separated character [46], which is related to a dark state acting as an intermediate step to the formation of two triplets (singlet fission, SF) [47]. This process has been reported in solutions of a pentacene derivative [48], which is a similar compound to FZ3. Furthermore, SF has been studied in pentacene solutions, single crystals, and pure films, through TA spectroscopy [48,49]; indeed, its TA features resemble those of FZ3. Actually, FZ3 has a small diradical character (*y*_0_ = 18%) [37], and SF has been theoretically predicted and experimentally demonstrated to occur in molecules with a small diradical character, such as zethrenes [50]. Then, we postulate that this negative band might correspond to a charge-separated state present in FZ3, which might lead to SF in concentrated solutions/films. Further studies will be aimed at corroborating this hypothesis.

### 3.3. DFB Lasers with FZ3-Doped Active Films and Top-Layer Resonator

**Figure 3:**
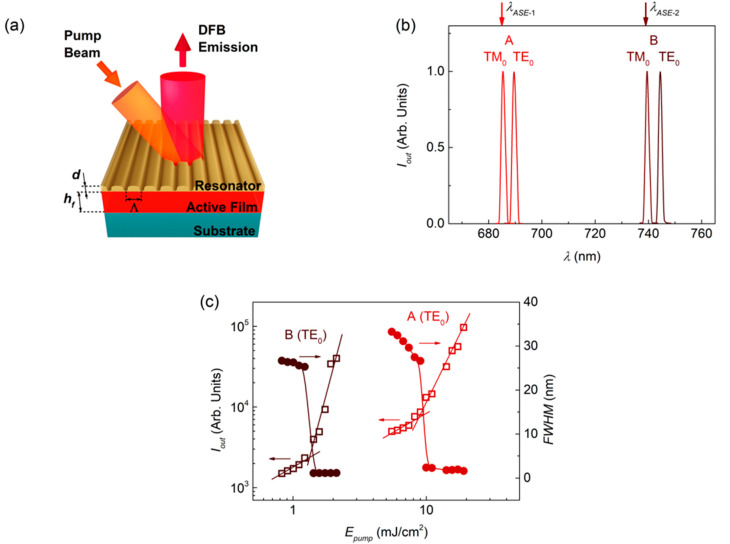
Distributed feedback (DFB) lasers based on 1D resonators. (**a**) Scheme of a DFB laser device with a 1D relief grating resonator of period Λ and depth *d* on top of the active film; (**b**) Emission spectra of DFB lasers based on FZ3-doped PS films: A (red peaks) and B (brown peaks) correspond to devices designed to work close to *λ_ASE_*_-1_ and *λ_ASE_*_-2_, respectively. Each laser mode is associated to a given waveguide mode (TE_0_ or TM_0_), as indicated next to each peak; (**c**) Output intensity (open squares) and emission line width, defined as the full width at half maximum *FWHM* (full circles), as a function of the pump energy density, *E_pump_*, for the laser peaks associated to the TE_0_ mode, for devices A (red) and B (brown). Full lines are guides to the eye.

**Figure 4:**
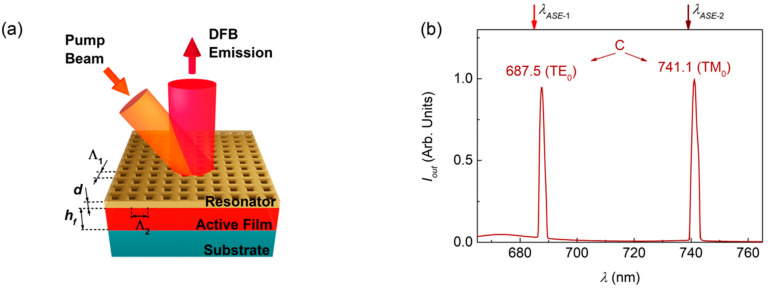
DFB laser based on a 2D resonator. (**a**) Scheme of a DFB laser device with a square 2D relief grating with periods Λ_1_ and Λ_2_ and depth *d*; (**b**) Laser spectrum for device C, showing two laser peaks at wavelengths (see values next to each peak) close to *λ_ASE_*_-1_ = 685 and *λ_ASE_*_-2_ = 739 nm.

The existence of dual ASE in FZ3-doped films is used here to fabricate two types of DFB lasers: (1) with emission corresponding to one of the two possible ASE wavelengths, *λ_ASE_*_-1_ = 685 nm or *λ_ASE_*_-2_ = 739 nm (devices A and B, respectively; see Figure 3 and Table 2); and (2) with emission corresponding to both ASE wavelengths simultaneously (device C, see Figure 4 and Table 2).

In all cases, we used resonators consisting of surface relief gratings, recorded by HL and subsequent dry etching, over water-processed DCG layers that had been previously deposited over the active films (see Section 2.2) [42,51]. For the first type DFB lasers, we used 1D gratings (see device Scheme Figure 3a). On the other hand, for the second type of devices, square 2D gratings were used, which can be envisaged as two 1D gratings perpendicular to each other (see the device scheme in Figure 4a).

There are various distinctive features of the architecture used in these devices (with the resonator in a separate layer located above the active film), which provide advantages with respect to others [51]. Firstly, the DCG layer is processed from water. So, its deposition over the active film, which is only soluble in organic solvents, does not distort the active film properties. Besides, the larger device size (cm) and the versatility offered by HL enable the preparation of devices emitting at different wavelengths, just by recording gratings of different periods in the same film. Moreover, because the active film thickness (*h_f_*) is uniform across the device, a low threshold is maintained for the whole emission wavelength range. All the 1D gratings prepared have been designed to operate in the second order of diffraction, which is *m* = 2 in the Bragg condition (Equation (2)) [1,52,53]:

(2) $$m\;\lambda_{\mathrm{Bragg}}=2\;n_{\mathrm{eff}}\;\Lambda ,$$

where *n_eff_* is the effective refractive index of the waveguide (which depends on *h_f_* and on the refractive indexes of film, substrate, and cover), and Λ is the grating period. This latter parameter is adjusted to obtain *λ_Bragg_* close to *λ_ASE_*, at which the gain is maximum, which is important to optimize the laser threshold. Considering light traveling in a given waveguide mode (for the present case, either the TE_0_ or TM_0_), predictions from coupled mode theory [54] indicate the appearance of a photonic stop-band (a dip) centered at *λ_Bragg_*. Thus, lasing would oscillate on a pair of wavelengths: one at either edge of the stop-band. However, in second-order devices, single-mode emission at the peak of the longer wavelength (*λ_DFB_*) is observed, because of the larger threshold of the other peak due to radiation losses [55]. Note that these predictions correspond to systems with pure index gratings, which is precisely the case here, because *h_f_* is constant, and the grating is in a separated layer.

Laser spectra for devices A and B (type 1) are shown in Figure 3b. They had corresponding gratings with periods of 446 and 481 nm, which are appropriate to obtain emission close to *λ_ASE_*_-1_ and *λ_ASE_*_-2_, respectively. As seen in Figure 3b and Table 2, both lasers show two peaks (the laser modes). Each of these peaks is associated to a given mode of the waveguide, which for the present case can be either the TE_0_ or TM_0_. The correlation between laser modes and waveguide modes can be done by looking at the polarization of the emitted light (TE or TM if light is polarized parallel or perpendicular to the grating lines, respectively). It can also be corroborated through calculations of *n_eff_* (by a software program such as *1*-*D mode solver for dielectric multilayer slab waveguides*) [56] and then, of *λ_Bragg_* through Equation (2). As seen in Figure 3b and Table 2, both devices A and B show two peaks, whose *λ_DFB_* values are close to the corresponding *λ_ASE_* value in each case. For both, the peak associated to the TM_0_ mode appears at a wavelength practically coincident with *λ_ASE_*, and the peak associated to the TE_0_ mode appears a few nm above. Note that the threshold for the latter is lower, despite it being further away from *λ_ASE_*. This can be explained by the lower losses of modes with TE polarization. In fact, in many organic DFB lasers reported in the literature, only the modes with TE polarization are observed, because their thresholds are lower [42,51,57]. It should also be noted that the TE_0_ peak with the lowest threshold (1.3 mJ·cm^−2^) is the one of device B, occurring close to *λ_ASE_*_-2_, particularly at 744.5 nm. This is in agreement with the lower ASE threshold observed at *λ_ASE_*_-2_, in comparison to the one at *λ_ASE_*_-1_.

The laser spectrum for device C (type 2) is shown in Figure 4b. The grating periods in each direction were 442 and 482 nm, which were designed to obtain emission close to *λ_ASE_*_-1_, and *λ_ASE_*_-2_, respectively. The most remarkable result is the possibility to obtain laser emission simultaneously at two different wavelengths (687.5 and 741.1 nm). The two observed peaks, which are separated by around 50 nm, are associated to the existence of dual ASE. Note that in this device, only one peak at each ASE wavelength is observed, instead of the two expected associated to TE_0_ and TM_0_, as it occurs in devices A and B. In device C, the laser peak close to *λ_ASE_*_-1_, (685 nm) appears at 687.5 and has TE polarization. There is no visible peak associated to the TM_0_ mode, which would be expected a few nm below *λ_ASE_*_-1_, which is most likely because its threshold would be rather large. On the other hand, the laser peak appearing close to *λ_ASE_*_-2_ (739 nm) is seen at 741.1 nm, and it has TM polarization. In this case, the peak associated to the TE_0_ mode would be expected at a longer wavelength, thus significantly deviated from *λ_ASE_*_-1_, and therefore with a large threshold, which would justify why it is not observed. Further work with a larger set of 2D devices is needed to reach a further understanding of these effects. This is out of the scope of the present study, whose purpose is to demonstrate the possibility to obtain lasers with dual emission associated to two ASE peaks of the same material.

## 4. Conclusions

In summary, dual ASE has been reported in FZ3-doped PS films. The two observed ASE bands are centered at around 685 and 739 nm, and they correspond respectively to gain bands associated to the main transition (0′→0) and its vibronic replica (0′→1), as it has been elucidated from the ∆*α/α* spectrum. The existence of two gain bands of similar intensity in FZ3 is attributed to a negative broad band observed in its TA spectrum. The latter possibly related to a charge-separated state; it equilibrates the gain band associated to the vibronic replica with that associated to the main transition by partially canceling the former. The suitability of both gain bands for gain applications is demonstrated by the fabrication of two types of DFB lasers, which are designed to emit either at a wavelength close to one of the ASE bands or simultaneously at the two ASE bands.

## Figures and Tables

**Table 1:** Optical and ASE parameters for FZ3-doped PS films. NG: nanographenes.

NG ^1^	*λ_ABS_*^2^ (nm)	*λ_PL_*^3^ (nm)	*τ_PL_*^4^ (ns)	*λ_pump_*^5^ (nm)	*σ_SE_*^6^ (10^16^ cm^2^)	*λ_ASE_*^7^ (nm)	*FWHM*^8^ (nm)	*E_th-ASE_*^9^ (mJ·cm^−2^)
FZ3	668/613	676/740	3.1 ± 0.3	613	5.7	685	3	60
-	-	-	-	-	-	739	6	4.4

^1^ The device consists of an active film of polystyrene doped with 1 wt % (error approximately 0.1%) of nanographene on top of a quartz substrate; ^2^ Wavelengths of main absorption peaks (the underlined value corresponds to the most intense one); ^3^ Wavelengths of main photoluminescence peaks (the underlined value corresponds to the most intense one); ^4^ Photoluminescence lifetime; ^5^ Pump wavelengths for the ASE experiments; ^6^ Stimulated emission cross-section (error: approximately 20%); ^7^ ASE wavelength (error ±0.5 nm); ^8^ ASE linewidth (error ±1 nm) defined as the full width at half maximum, well above the threshold; ^9^ ASE threshold, expressed as energy density (error: approximately 20%).

**Table 2:** Parameters for FZ3-based DFB lasers.

Device ^1^	Grating ^2^	Λ ^3^ (nm)	*λ_ASE_*^4^ (nm)	*λ_DFB_*^5^ (nm)	*E_th-DFB_*^6^ (mJ·cm^−2^)
A	1D	446	685	685.4 (TM_0_)	15
-	-	-	-	689.5 (TE_0_)	9.5
B	1D	481	739	739.5 (TM_0_)	14
-	-	-	-	744.5 (TE_0_)	1.3
C	2D	442/482	685/739	687.5 (TE_0_)/741.1 (TM_0_)	8.7/7.2

^1^ The DFB device consists of an active film of polystyrene doped with 1 wt % (error approximately 0.1%) of nanographene with a top-layer of dichromated gelatine with an engraved relief grating; ^2^ Dimensionality of the engraved grating; ^3^ Grating period (error approximately 0.5%); ^4^ Nearest ASE wavelength to the DFB emission; ^5^ DFB wavelength (error is ±0.5 nm) of the laser mode and associated waveguide mode in brackets. The emission is polarized parallel (TE_0_) or perpendicular (TM_0_) to the DFB grating lines; ^6^ DFB threshold (error approximately 10%), expressed as energy density.

## Supplementary Materials

The following are available online at https://www.mdpi.com/2079-4991/10/8/1525/s1, Figure S1: Transient absorption spectra for FZ1-doped PS films, Figure S2: Transient absorption spectra for FZ2-doped PS films, Figure S3: Time-resolved PL for FZ-doped PS films, Table S1: Transient absorption parameters for FZ-doped PS films.
