# Supplementary material for: Dual Amplified Spontaneous Emission and Lasing from Nanographene Films
Source: Nanomaterials (Basel). 2020 Aug 4;10(8):1525. doi: 10.3390/nano10081525 (PMC7466616; doi:10.3390/nano10081525)
Supplement: Supplementary file 1 [file nanomaterials-10-01525-s001.pdf]

Supporting Information

Dual Amplified Spontaneous Emission and Lasing from Nanographene Films

Rafael Muñoz-Mármol [1], Víctor Bonal [1], Giuseppe M. Paternò [2], Aaron M. Ross [3], Pedro G. Boj [4], José M. Villalvilla [1], José A. Quintana [4], Francesco Scotognella [2,3], Cosimo D'Andrea [2,3], Samim Sardar [2], Guglielmo Lanzani [2,3,*], Yanwei Gu [5], Jishan Wu [5,*] and María A. Díaz-García [1,*]

[1] Departamento de Física Aplicada and Instituto Universitario de Materiales de Alicante, Universidad de Alicante, Alicante 03080, Spain; rafa.marmol@ua.es (R.M.-M.); victor.bonal@ua.es (V.B.); jmvs@ua.es (J.M.V.);

[2] Center for Nano Science and Technology, Istituto Italiano di Tecnologia, Via G. Pascoli 70/3, Milano 20133, Italy; giuseppe.paterno@iit.it (G.M.P.); francesco.scotognella@polimi.it (F.S.); cosimo.dandrea@polimi.it (C.D.A.); Samim.Sardar@iit.it (S.S.)

[3] Physics Department, Politecnico di Milano, Piazza L. da Vinci 32, Milano 20133, Italy; aaronmichael.ross@polimi.it

[4] Departamento Óptica, Farmacología y Anatomía and Instituto Universitario de Materiales de Alicante, Universidad de Alicante, Alicante 03080, Spain; p.boj@ua.es (P.G.B); ja.quintana@ua.es (J.A.Q.)

[5] Department of Chemistry, National University of Singapore, 3 Science Drive 3, Singapore 117543, Singapore.

* Correspondence: guglielmo.lanzani@iit.it (G.L.); chmwuj@nus.edu.sg (J.W.); maria.diaz@ua.es (M.A.D.-G.)

Supporting 1. Transient absorption for FZ1

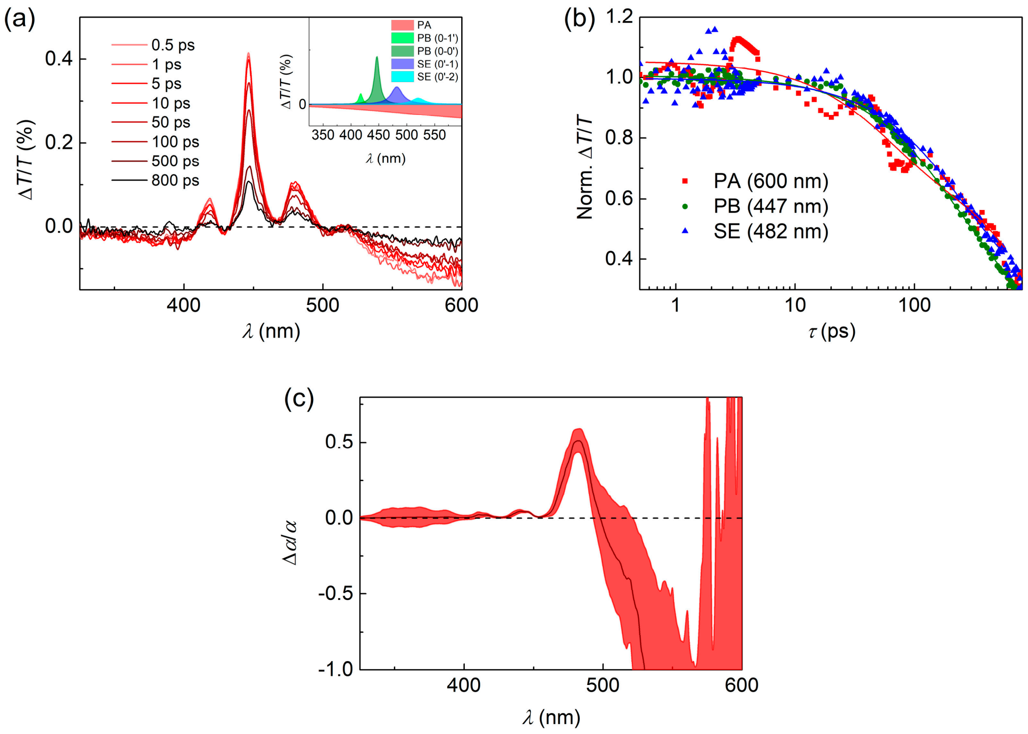

Figure S1. Transient absorption spectra for FZ1-doped PS films. (**a**) TA spectra obtained at a pump wavelength of 390 nm and energy density of 240 μJ/cm^2, for a series of pump-probe delays (see legend). The inset represents the deconvolution of the 10 ps pump-probe delay curve into five different contributions, attributed to photobleaching (PB), photoinduced absorption (PA) or stimulated emission (SE); (**b**) Transient dynamics for the peaks at 600 nm, 447 nm and 482 nm attributed to PA, PB and SE peaks, respectively. Solid lines correspond to double exponential fits to the data; (**c**) Pump induced absorption coefficient normalized to steady state absorption coefficient (dark red line) and its error estimation (red area).

Supporting 2. Transient absorption for FZ2

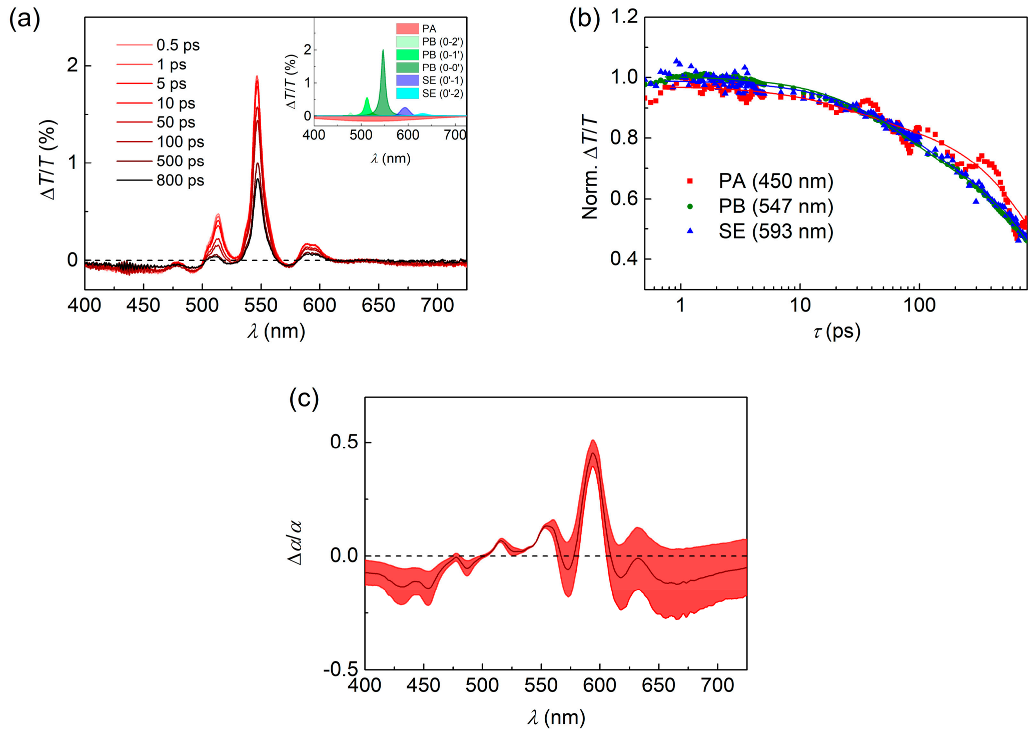

Figure S2. Transient absorption spectra for FZ2-doped PS films. (**a**) TA spectra obtained at a pump wavelength of 507 nm and energy density of 16 μJ/cm^2, for a series of pump-probe delays (see legend). The inset represents the deconvolution of the 10 ps pump-probe delay curve into six different contributions, attributed to photobleaching (PB), photoinduced absorption (PA) or stimulated emission (SE); (**b**) Transient dynamics for the peaks at 450 nm, 547 nm and 593 nm attributed to PA, PB and SE peaks, respectively. Solid lines correspond to double exponential fits to the data; (**c**) Pump induced absorption coefficient normalized to steady state absorption coefficient (dark red line) and its error estimation (red area).

Supporting 3. Time-resolved Photoluminescence

The PL dynamics of FZ1 was measured by the time-resolved PL system based on the streak camera, while that of FZ2 and FZ3 were obtained with the one based on TCSPC detection. Both setups have been described in the method section of the main paper.

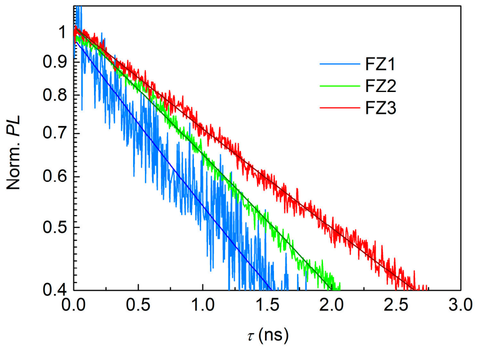

Figure S3. Time-resolved PL for FZ-doped PS films. Normalized output intensity at the first emission peak for PS films doped with FZ1 (blue), FZ2 (green) and FZ3 (red), as a function of time. Solid lines correspond to decay exponential fits to the experimental data.

Supporting Table 1. Transient absorption parameters.

Table 1. Transient absorption parameters for FZ-doped PS films.

NG[1]	h[2] (nm)	λ_{Abs}[3] (nm)	λ_{PL}[4] (nm)	τ_{fl}[5] (ns)	λ_{pump}[6] (nm)	A_{pump}[7] ($\cdot 10^{-4}$ cm^2)	E_{pump}[8] ($\mu J \cdot cm^{-2}$)	$\alpha[\lambda_{pump}]$[9] ($\cdot 10^3$ cm^{-1})	σ_{SE}[10] ($\cdot 10^{-16}$ cm^2)	τ_{TA}[11] (ns)
FZ1	410	452/426/402	455/485/519	1.6±0.2	390	5.3	240	0.37	≈2	1.0/0.1
FZ2	480	545/509/474	547/588/636	2.14±0.04	507	11	16	1.70	7.5	1.5/0.06
-	-	-	-	-	546	8.6	21	4.44	8.5	0.9/0.07
FZ3	600	668/613	673/732	3.1±0.3	618	15	30	0.69	5.7	1.0/0.05
-	-	-	-	-	667	23	26	4.10	3.9	1.0/0.08

[1]The device consists of an active film of polystyrene doped with 1 wt% (error~0.1%) of nanographene on top of a quartz substrate. [2]Film thickness (error~2%). [3]Wavelengths of main absorption peaks (the underlined value corresponds to the most intense one). [4]Wavelengths of main photoluminescence peaks (the underlined value corresponds to most intense one). [5]Photoluminescence lifetime. [6]Pump wavelengths for the TA experiments. [7]Pumped area for the TA experiments (error 10%). [8]Pump fluence (error 20%). [9]Absorption coefficient at λ_{pump} (error~20%). [10]Stimulated emission cross-section (error~20%). [11]Transient absorption life-time (error~20%); the first value corresponds to the dominant component.
